# Supplementary material for: Survival Analysis of Hospitalized Elderly People with Fractures in Brazil Over One Year
Source: Geriatrics (Basel). 2020 Feb 19;5(1):10. doi: 10.3390/geriatrics5010010 (PMC7151474; doi:10.3390/geriatrics5010010)
Supplement: Supplementary file 1 [file geriatrics-05-00010-s001.pdf]

Table S1.

ICD	BASIC CAUSE	TOTAL	
		**N°**	**%**
W18	Other fall on same level	38	34,5
W01	Fall on same level from slipping, tripping and stumbling	6	7,0
V03	Pedestrian injured in collision with car, pick-up truck or van	4	4,7
V18	Pedal cyclist injured in noncollision transport accident	3	3,5
V23	Motorcycle rider injured in collision with car, pick-up truck or van	4	4,7
A41.9	Sepsis, unspecified	1	1,2
J44.0	Chronic obstructive pulmonary disease with acute lower respiratory infection	2	2,3
C61	Malignant neoplasm of prostate	1	1,2
V18.4	Pedal cyclist injured in noncollision transport accident – driver injured in traffic accident	2	2,3
I67	Other cerebrovascular diseases	1	1,2
I70.9	Generalized and unspecified atherosclerosis	1	1,2
I21.9	Acute myocardical infaction, unspecified	1	1,2
W13	Fall from, out of or through building or structure	1	1,2
V69	Occupant of heavy transport vehicle injured in other and unspecified transport accidents	2	2,3
W08.0	Fall involving other furniture - residence	1	1,2
I50.9	Heart failure unspecified	1	1,2
N19	Unspecified kidney failure	1	1,2
I69	Sequelae of cerebrovascular disease	1	1,2
E14	Diabetes mellitus	4	4,7
E88.9	Metabolic disorder, unspecified	1	1,2
S06	Intracranial injury	1	1,2
C34	Malignant neoplasm of bronchus and lung	2	2,3
X59	Exposure to unspecified factor	1	1,2
I87.2	Venous insufficiency (chronic) (peripheral)	1	1,2
I10	Essential (primary) hypertension	1	1,2
R54	Senility	1	1,2
K74.6	Other and unspecidied cirrhosis of liver	1	1,2
B57.3	Chagas disease (chronic) with digestive system involvement	1	1,2
C22.9	Liver, unspecidied	1	1,2

ICD Internacional Disease Registration

Table S2.

ICD	END CAUSE	TOTAL	
		**N°**	**%**
J96.0	Acute respiratory failure	12	14,1
T79	Certain early complications of trauma, not elsewhere classified	9	10,6
T79.4	Traumatic shock	17	20,0
T81.1	Shock during or resulting from a procedure, not elsewhere classified	2	2,3
S06	Intracranial injury	4	4,7
S72	Fracture of femur	2	2,3
J18.9	Pneumonia, unspecified	1	1,2
A41.9	Sepsis, unspecified	8	9,4
R68.8	Other specified general symptoms and signs	4	4,7
J80	Adult respiratory distress syndrome	2	2,3
C78.0	Secondary malignant neoplasm of lung	1	1,2
T79.3	Post-traumatic wound infection, not elsewhere classified	1	1,2
I26.9	Pumonary embolism without mention of acute cor pulmonale	4	4,7
T07	Unspecified multiple injuries	2	2,3
T81.7	Vascular complications following a procedure, not elsewhere classified	1	1,2
R09.2	Respiratory arrest	2	2,3
R57.0	Cardiogenic shock	1	1,2
T79.0	Air embolism (traumatic)	2	2,3
I21.9	Acute myocardial infarction, unspecidied	2	2,4
R64	Cachexia	1	1,2
E14	Unspecified diabetes mellitus	1	1,2
I10	Essential (primary) hypertension	1	1,2
R96.0	Instantaneous death	1	1,2
R57	Shock, not elsewhere classified	2	2,3
R57.1	Hypovolaemic shock	1	1,2
C22.9	Liver, unspecified	1	1,2

ICD Internacional Disease Registration
